# Descriptive DNA barcoding of *Argas* (*Persicargas*) *arboreus* and *Argas* (*Persicargas*) *persicus* ticks (Ixodida: Argasidae) infesting birds in Egypt

**DOI:** 10.1007/s10493-022-00768-x

**Published:** 2022-12-02

**Authors:** Enas H. Ghallab, Ayat Yousery, Mona G. Shaalan

**Affiliations:** Entomology Department, Faculty of Science, Ain Shams University, Abbassia Square, El-Khalifa El-Maamoun Street, Cairo, 11566 Egypt

**Keywords:** *Argas*, COI, DNA barcoding, Egypt

## Abstract

*Argas* ticks are primary parasites of birds with veterinary importance. Nevertheless, these ticks have received little attention regarding molecular identification studies. DNA barcoding is a powerful technique for identifying tick species besides traditional morphological identification. The present work is a first effort to divulge DNA sequences of *Argas* (*Persicargas*) *arboreus* from Egypt and worldwide. We used cytochrome c oxidase subunit I (COI) from *A. arboreus* infesting herons, and from the fowl tick *Argas* (*Persicargas*) *persicus*. Our results pointed out another success for the Folmer primers that are widely used in DNA barcoding, permitting the discrimination of morphologically similar *A. arboreus* and *A. persicus*.

## Introduction

Ticks are ectoparasites of health concern. They vector a variety of pathogens such as *Babesia*, *Theileria*, *Rickettsia*, and *Borrelia* to humans and animals, ranking second after mosquitoes in disease transmission (Orkun et al. 2014; de la Fuente et al. 2008; Shah et al. 2004; Parola and Raoult 2001). Furthermore, ticks may cause direct damage to their hosts through attachment and feeding (de la Fuente et al. 2008; Pagel Van Zee et al. 2007). Argasid ‘soft’ ticks are multi-host blood-feeders occurring in the nests of birds and shelters of small mammals throughout temperate and tropical regions of the world, being of concern to agriculturists (Hoogstraal 1985; Cooley and Kohls 1944). *Argas* (*Persicargas*) *arboreus* is a common parasite of the cattle egret (*Bubulcus ibis*) and other water birds in Egypt and all over the African continent (Khalil et al. 1980; Kaiser et al. 1964). This bird benefits agriculturists by feeding on insect pests and tiny animals. In Egypt, dozens of breeding immature cattle egrets were found dead on the ground beneath heronries infested with *A. arboreus* (Hoogstraal 1985). Furthermore, *A. arboreus* in Egypt may host *Salmonella typhimurium* (Floyd and Hoogstraal 1956), *Wolbachia persica* (Suitor and Weiss 1961), and *Borrelia anserina* (Hoogstraal 1985). It is also naturally infected with Quaranfil and Nyamanini viruses (Hoogstraal 1966; Taylor et al. 1996). Bird migration contributes to the dispersal of such ticks and the transmission of diseases (Khalil et al. 1980).

Likewise, the fowl tick *Argas* (*Persicargas*) *persicus* is an ectoparasite of poultry birds, which causes severe irritation to poultry, harming their production (Shah et al. 2004; Phulan et al. 1984). Heavy infestations may cause blood loss, resulting in anemia and death (Alzahrani and Edrees 2020). These ticks also may transmit specific parasitic, bacterial, and viral agents, such as leucocytozoonosis, aegyptianellosis, pasteurellosis, avian encephalomyelitis, borreliosis, and fowl cholera (Permin and Hansen 1998), thus leading to heavy economic losses in the poultry industry.

*Argas arboreus* was morphologically confused with *A. persicus* until it was recognized as a new species by Kaiser et al. (1964). Indeed, morphological differences between these closely related tick species may be slight; but their capacity as vectors for pathogenic microorganisms may differ dramatically (Hoogstraal 1985). Therefore, accurate differentiation of such species is crucial (Abdullah et al. 2016; Jongejan and Uilenberg 1994). Morphological identification of ticks is limited by several factors, such as specimen integrity and tick developmental stage. Most keys rely on adult identification; thus, it is challenging to identify immature ticks using pictorial identification keys (Takano et al. 2014).

Molecular examinations have evolved to overcome inaccurate taxonomic identification. The recent explosion of biological information has offered a tool to construct a complete database for ticks, allowing information and proper identification of various tick species (Ghosh et al. 2007). Molecular barcoding provides a highly efficient alternative for identifying biological species and has been used extensively in the study of arthropod taxonomy (Castalanelli et al. 2014; Taylor and Harris 2012; Gariepy et al. 2007; Smith et al. 2005). A highly efficient molecular region is used to identify various arthropods, including ticks, butterflies and mosquitoes: cytochrome c oxidase subunit 1 (COI). According to Lv et al. (2014a, b), COI is the best genomic marker for DNA barcoding. The advantage of using COI is that it is a small enough fragment (about 648 bp long) to be sequenced using a cheap and quick technique.

Accordingly, we aimed to reveal DNA barcoding for the identification of *A. arboreus* and *A. persicus* in Egypt. Moreover, to establish the phylogenetic relationship of *A. arboreus* and *A. persicus* COI regions among different *Argas* species worldwide.

## Materials and methods

### Tick collection

In total 250 tick specimens were collected from two localities in Egypt. One hundred tick specimens were collected from trees supporting the rookeries of the cattle egret *Bubulcus ibis* et al. Mansoureya Canal, Giza governorate (30° 00′ 26.9′′ N, 31° 06′ 58.9′′ E). At the same time, 150 tick specimens were collected from domestic chicken houses in the Abo-Rawash, Giza governorate (30° 02′ 36.1′′ N, 31° 05′ 58.8′′ E). The collected ticks were transferred to the laboratory, morphologically identified according to Kaiser et al. (1964), and maintained under laboratory conditions at 28 ± 2 °C for further analysis.

### Extraction of genomic DNA from ticks

Freshly collected ticks were morphologically identified then stored at − 20 °C. For further molecular analysis, individual ticks were washed with cold ethanol and their DNA was extracted using the DNeasy Blood & Tissue extraction mini kit (cat. no. 69504; Qiagen, Hilden, Germany), according to the manufacturer’s protocol.

### COI amplification

The COI gene was amplified using Folmer universal primers LCO1490 (GGTCAACAAATCATAAAGATATTGG) and HCO2198 (TAAACTTCAGGGTGACCAAAA AATCA) (Folmer et al. 1994). The reaction components of 20 µl total volume were: ×1 PCR buffer, 200 µM each of deoxynucleotide triphosphate (dNTPs), 0.5 unit of Taq polymerase (HotStar Taq DNA Polymerase, Qiagen), and 3.2 pmol each of the forward and reverse primers. A gradient PCR assay with different annealing temperatures ranging from 55 to 65 °C for 40 cycles was developed to optimize the PCR assay. PCR products were visualized on an 1.5% agarose gel containing ethidium bromide (0.375 µg/ml). Hyperladder II (Bioline) was used as a DNA marker. The optimum thermocycling reaction conditions were as follows: one cycle at 94 °C for 30 s for the initial denaturation, 40 cycles of denaturation at 94 °C for 30 s, annealing at 48 °C for 30 s, extension at 72 °C for 60 s, and a final extension cycle at 72 °C for 10 min.

### Direct sequencing

Purified PCR products of COI were bidirectly sequenced by the Sanger dideoxy sequencing method (Sanger et al. 1977) as follows: denaturation from 1 to 96 °C, then 25 cycles of 30 s at 96 °C, annealing at 50 °C for 15 s, and an extension at 60 °C for 4 min. Resulting DNA sequence chromatograms were analyzed and assembled into a single contig. For conferring sequence identity, every single sequence was pairwise aligned against homologous sequences in the GenBank database using the NCBI BLASTn (Zhang et al. 2000).

## Results

**Fig. 1:**
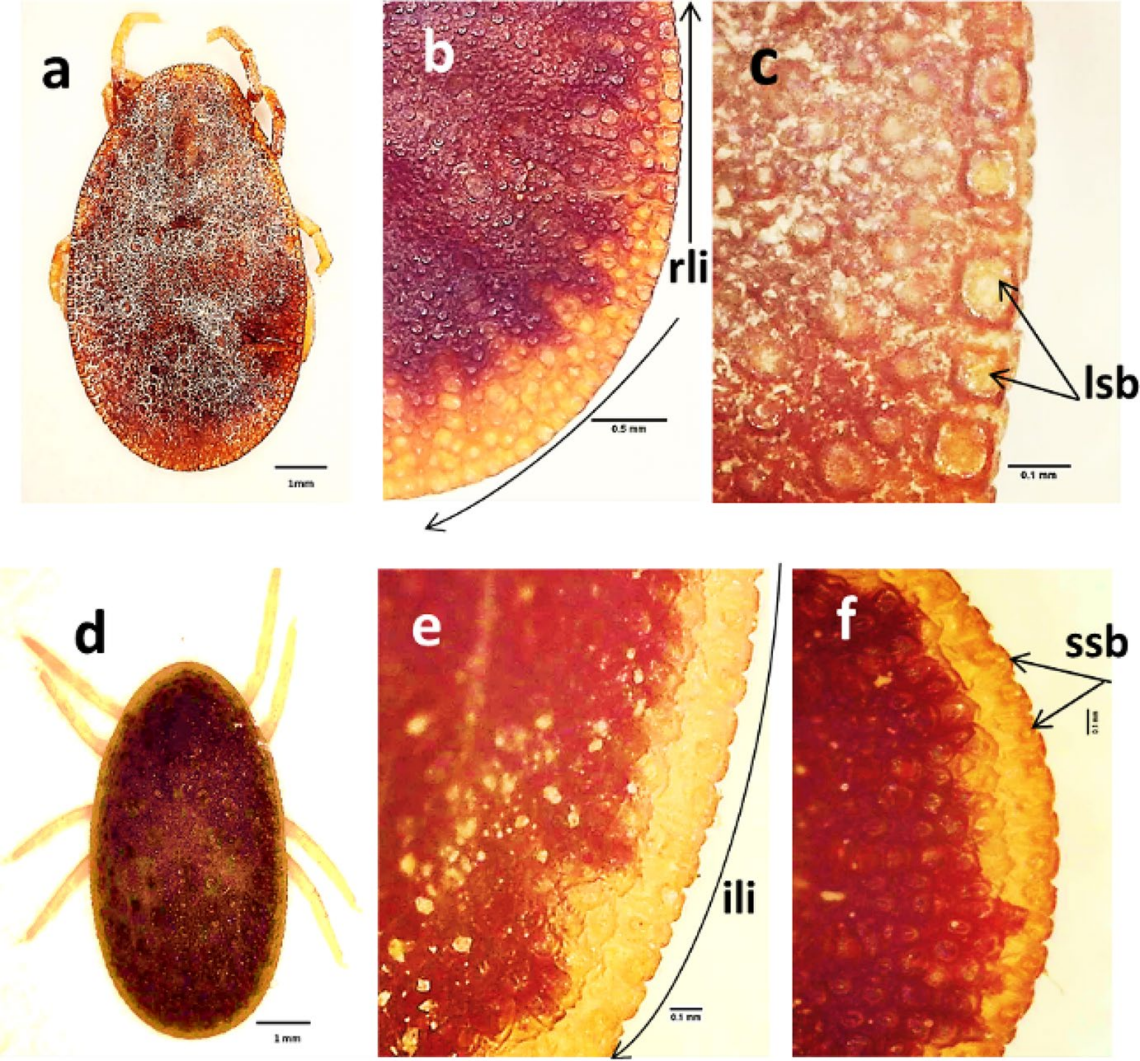
Differential morphology of *Argas* (*Persicargas*) *persicus* and *A.* (*P.*) *arboreus.* **a** Dorsal view of *A.* (*P.*) *persicus* female. **b**, **c** Regular lateral integumental cells (rli) contain larger setae bearing pit (lsb). **d** Dorsal view of *A.* (*P.*) *arboreus* female. **e**, **f** Irregular lateral integumental cells (ili), each containing small setae bearing pit (ssb)

Microscopical examination of ticks (n = 100) collected from trees supporting the rookeries of the cattle egret *B. ibis* were *A*. *arboreus* (42 females, 23 males, and 35 nymphs). Ticks (n = 150) collected from domestic chicken houses were *A. persicus* (57 females, 45 males and 48 nymphs). Collected ticks were morphologically differentiated by the lateral integumental cells (Kaiser et al. 1964). *Argas arboreus* had irregular lateral integumental cells, each containing small setae bearing pit. In *A. persicus* such cells were larger, fewer in number, had a more regular outline, and contained considerably larger setae bearing pit (Fig. 1).

For each species identified, 15 specimens were submitted to DNA extraction and PCR amplification. Mitochondrial COI region of nearly all involved samples were successfully PCR amplified—13 samples were successfully amplified for *A. arboreus*, and 14 for *A. persicus*, with the expected PCR product of 710 bp. However, due to technical issues, we successfully amplified and trimmed the sequences to 603 and 573 bp from *A*. *arboreus* and *A. persicus*, respectively.

Resulting sequences were searched against the BLAST database (Zhang et al. 2000) and BOLD system (Ratnasingham and Hebert 2007). No previous report of amplified reference for *A. arboreus* sequenced gene was retrieved. When searched against BLASTn, the highest similarity scores were 90.5% (*Argas miniatus* mitochondrion, complete genome, KC769590.1), 85.1% (*A. persicus* clone Contig35_14411_396 mitochondrion, complete genome, KJ133581.1), and 84.8% (*Argas walkerae* clone Contig172/296/583_14437_314 mitochondrion, complete genome, KJ133584.1).

On the other hand, for *A. persicus*, BLASTn highest similarity scores were 98.2% (*A. persicus* clone Contig35_14411_396 mitochondrion, complete genome, KJ133581.1), 98.4% (*A. persicus* isolate 95-6-13 COI gene, partial cds; mitochondrial, KX879770.1), and 97.8% (*A. persicus* mitochondrial partial COI gene, isolate ASS, FN394341.1). Sequences presently revealed have been deposited in the GenBank database with accession numbers OM177656 [*A. arboreus* COX1] gene and OM177661 [*A. persicus* COX1] gene.

Multiple sequence alignment of the COI gene nucleotide sequence for *A. persicus*, OM177661, against *A. arboreus* OM177656 revealed 76.28% sequence identity. Phylogenetic analysis was established based on COI sequences of as many *Argas* species as available on the GenBank. A neighbor-joining tree of the COI gene from *Argas* species worldwide was constructed using MEGA11 (Tamura et al. 2021). The substitutions type was set to the nucleotide with kimura 2-parameter model and bootstrap 1000 replicates. This analysis involved 23 nucleotide sequences as follows: *A. persicus* ‘Egypt, current study (OM177661); Kenya (KJ133581.2); Romania (FN394341); USA (MK287892.1); Iran (KX879770.1), Pakistan (MW077849); Kazakhstan (MN900726) and China (LC209195)’; *A. arboreus* COI (OM177656) ‘Egypt, current study’; *Argas* sp. Spain (MW288380, MW288384, MW288385, MW288386, MW288387 (Palomar et al. 2021); *A.* (*Secretargas*) *transgariepinus* South Africa (KX431961); *A. africolumbae* South Africa (NC_019642); *A. lagenoplastis* Australia (KC769587); *A.* (*Carios*) *vespertilionis* Vietnam (KX431960); *A. miniatus* Brazil (KC769590); *A. reflexus* Spain (MW288388); *A. walkerae* South Africa (KJ133584); *A. hermanni* Iran (ON090294). In the constructed tree, *Dermanyssus* sp. (FN650615) was used as an out-group.

**Fig. 2:**
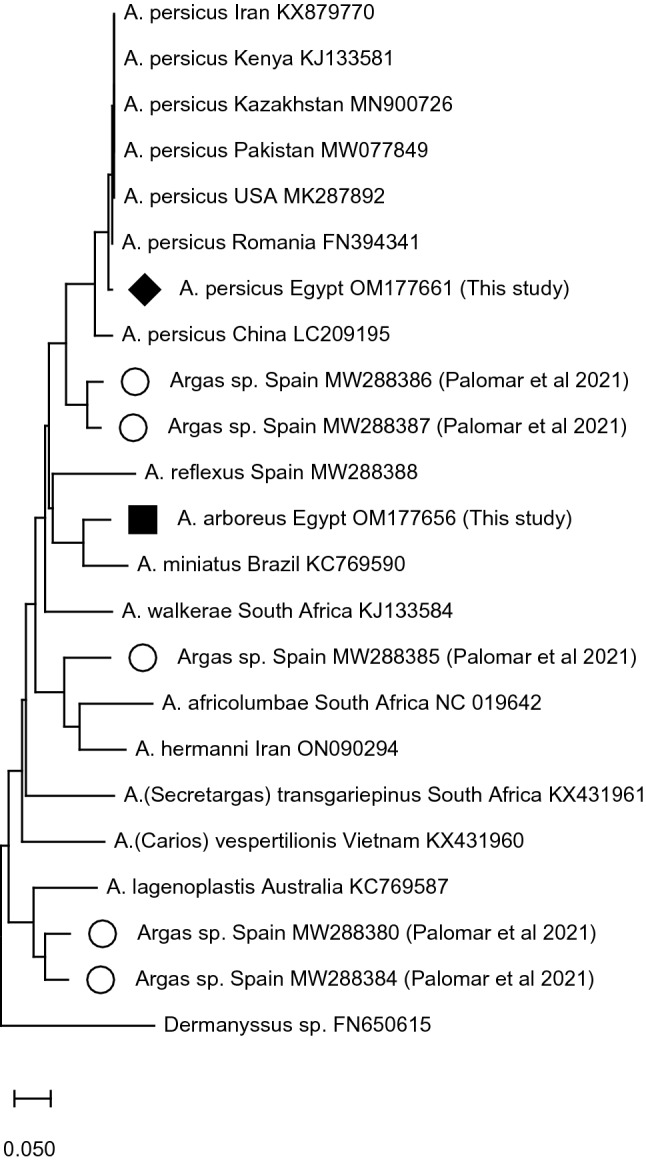
Neighbor-joining tree of the *Argas persicus* COI gene from different countries was constructed using MEGA11 (Tamura et al. 2021) with bootstrap values (1000 replicates). The tree was drawn to scale, with branch lengths in the same units as those of the evolutionary distances used to infer the phylogenetic tree. This analysis involved 23 nucleotide sequences as shown on the tree

The constructed NJ-phylogenetic tree (Fig. 2) demonstrated that all *A. persicus* groups, including *A. persicus* from collected specimens, were monophyletic and shared the same clade with haplotypes of *Argas* sp. from Spain, conferring their relatedness. Another result is the uniqueness of *A. arboreus* genotype in the present study. Indeed, such genotype shares the same molecular basis with *A. miniatus* as reported by Muñoz-Leal et al. (2018)*.* Furthermore, the same molecular basis was shared between *A. africolumbae* and *A. hermanni* as reported by Hoogstraal et al. (1975).

## Discussion

This study represents the first effort to reveal the DNA sequence of *A. arboreus* from Egypt and worldwide, and the sequence was the first mentioned on the GenBank. Despite the economic and veterinary impact of argasids, most studies of public and veterinary health have focused on hard ticks (Ixodidae) (Lv et al. 2014a, b). This study focused on two species of soft ticks (Argasidae) infesting birds in Egypt. Morphological identification of these two soft tick species showed that both belonged to the subgenus *Persicargas*.

In this study, ticks collected from trees supporting the rookeries of the cattle egret *B. ibis* were morphologically identified as *A. arboreus*. This species is known to infest herons and other medium-wading birds in many African areas (Khalil et al. 1980). Belozerov et al. (2003) studied the relationship between *A. arboreus* and heron life cycles. They found a seasonal synchronization between tick development and reproduction with the nesting and breeding periods of the birds during the spring and summer season in Egypt. Adult and nymphal stages of *A. arboreus* enter a diapausing period during the winter, during the migration of their hosts. This process lasts until the birds return to their heronries (Guirgis 1971).

Ticks collected from chicken houses in this study were morphologically identified as *A. persicus.* Poultry birds, such as chicken and turkey, are the primary hosts of *A. persicus* (Hoogstraal 1985). This tick is distributed worldwide, especially in tropical and subtropical regions of the world, and is considered a vector of avian spirochetosis. It can spread viruses and is the natural reservoir host of numerous infectious agents (Hoogstraal 1966, 1985). Kaiser et al. (1964) identified *A. arboreus* as a new species previously confused with *A. persicus*. Therefore, accurately identifying soft tick species is essential to developing a suitable strategy for minimizing the risk of damage to domestic fowls.

Only expert *Argas* taxonomists can comfortably identify and differentiate between closely related species. DNA barcoding enables non-taxonomists to identify closely related vectors with exceedingly difficult morphometrics. Therefore, we developed an effective barcode assay allowing non-experts to identify the tick species rapidly. DNA barcoding depends on a standard fragment of mitochondrial COI to successfully distinguish between closely related species (Zhang and Zhang 2014). Accurate and fast identification of vectors is extremely critical in vector-borne disease surveillance programs, especially for tick-related species of species complexes (Besansky et al. 2003).

The COI gene is an informative molecular marker on several taxonomic scales, particularly at the species level (Waugh 2007). The COI is considered one of the most useful DNA markers used for the molecular characterization of ticks (Yavari et al. 2019). Lv et al. (2014a, b) are considered the primer team focused on using DNA barcoding in the molecular identification of ticks. Instead of using only the COI gene, they relied on a three-gene DNA barcode system, including COI, 16S, and 18S. The use of a three-gene system was justified, not because species are more precisely delineated by it, but because some tick species lack a substantial COI sequence library on the BOLD system or GenBank to give species level matches. This occurred with our data available for *A. arboreus* with no available data neither on GenBank nor on the BOLD system and with a limited number of data available for *A. persicus* COI.

Our study affords a good potential technique that is compatible with the results of Gou et al. (2018) and Ondrejicka et al. (2017) for the identification of species in the Argasidae family, using the COI gene as part of an integrative approach combining mitochondrial gene markers and morphological characters.

In Iran a study was conducted on sequencing the COXI gene from eight samples from five provinces belonging to *A. persicus* (Yavari et al. 2019). It was found that all isolates had similar interspecific nucleotides, except for only one isolate that was similar to a specimen of *A. persicus* from South Africa and Romania. This result agrees with our sequence result for *A. persicus*, showing high-sequence similarity with the representatives from Romania.

According to Mans et al. (2019), the completion of the use of tick mitochondrial genes will be an area of future interest for tick systematics. Their study improved those mitochondrial genomes useful for studying species and higher-level relationships in ticks. Although representatives of most tick genera have been collected, several important genera are still missing, and some other species, such as *A. arboreus*, were determined there in this study.

Presently, the traditional taxonomy of the two species, *A. persicus* and *A. arboreus*, was supported by BLASTn analysis and the phylogenetic analysis of the mitochondrial COI nucleotide sequences. BLASTn analysis of the amplified COI nucleotide sequence of *A. persicus* showed 98% sequence identity to COI sequences of *A. persicus* from Kenya of the same species, with a high similarity percentage. The highest similarity percentage of the amplified COI sequence of *A. arboreus* was 90% with *A. miniatus* and this was explained through the phylogenetic tree as well. The relatedness between *A. arboreus.* and *A. miniatus* through sharing their molecular composition was reported by Muñoz-Leal et al. (2018), who used the mitochondrial 16S rRNA sequence gene and showed a 99–100% similarity between *A.* (*P.*) *robertsi* from Australia and *A.* (*P.*) *miniatus* from Brazil. Similarly, *A.* (*P.*) *robertsi* is considered to be the Oriental Australian counterpart of the Ethiopian *A.* (*P.*) *arboreus* (Hoogstraal et al. 1974).

According to Khalil et al. (1980), the African *A.* (*P.*) *arboreus* and the Asian-Australian *A.* (*P.*) *robertsi* originated from a common ancestor and geographic isolation caused genetic incompatibility. The present analysis helped reveal the significant genetic divergence between the two species in this study. Our phylogenetic analysis assured the uniqueness of the molecular marker COI in *A. arboreus* and its validity to be used in the differentiation process between the other species. In a try to assign the five sequences identified by Palomar et al. (2021) to the Geneus level, we blended them with our collected sequences retrieved from the GenBank. Two genotypes ‘MW288386 and MW288387’ were clustered with *A. persicus*, one genotype ‘MW288385’ was clustered with both *A. africolumbae* and *A. hermanni*, two genotypes ‘MW288380 and MW288384’ were clustered with *A. lagenoplastis*, all of these mentioned clustered in the tree in this study, supporting the sequence identities shown in Palomar et al. (2021), as well as represents a similar tree to the 16S rRNA analysis performed in Palomar et al. (2021)

## Conclusions

This study provides the first molecular descriptive barcoding of the two soft tick species that are major bird ectoparasites, especially on herons and poultry from the Giza governorate in Egypt, *A. arboreus* and *A. persicus*. A large-scale multi-marker study with a large sample size from different localities of soft ticks covering various hosts and populations across Egypt in different seasons is needed in the future. Such a study will provide a better and broader understanding of the population structure and dynamics, host range, physical environment, and habitat of these important medical and veterinary species. Additionally, their economic importance and effects on public health throughout the country will undoubtedly be better understood.

## Data Availability

COI sequences amplified in the present work were published in the GenBank under the Accession Numbers OM177661 and OM177656.
